# Evolutionary analysis and population dynamics in the global transmission of Kaposi’s sarcoma-associated herpesvirus

**DOI:** 10.1007/s00705-025-06259-9

**Published:** 2025-03-27

**Authors:** Tianye Wang, Jiaqi Wu, So Nakagawa, Takahiro Yonezawa, Zhenqiu Liu, Xin Zhang, Haili Wang, Yi Li, Tiejun Zhang

**Affiliations:** 1 Department of Epidemiology, School of Public Health, Fudan University, Shanghai, 200032 China; 2 Department of Molecular Life Science, Tokai University School of Medicine, Isehara, Kanagawa 259-1193 Japan; 3 Graduate School of Integrated Sciences for Life, Hiroshima University, Higashi-Hiroshima, 739-8528 Japan; 4 State Key Laboratory of Genetic Engineering, Human Phenome Institute, and School of Life Sciences, Fudan University, Shanghai, 200438 China

## Abstract

With the widespread prevalence of Kaposi’s sarcoma-associated herpesvirus (KSHV) globally, particularly in sub-Saharan Africa and the Mediterranean region, the open reading frame (ORF) K1 gene, a key gene for distinguishing different subtypes of KSHV, has been extensively studied for its diversity and sequence variations. In this study, we collected K1 gene sequences representing subtypes of KSHV worldwide in order to assess the global distribution of KSHV subtypes and to investigate the recombination and selection history of KSHV. Recombination and gene flow analysis indicated a minimum average recombination rate of 0.41 per site for the K1 gene. Recombination analysis indicated that 11 major recombination events had occurred, predominantly in subtypes A and C, while subtype B showed minimal involvement in recombination processes, consistent with the gene flow analysis. Using tip-dating methods, we estimated that the most recent common ancestor of KSHV emerged in the 12th century, while the currently globally prevalent subtypes appeared within the past three centuries. Its recent origin and rapid evolution indicate that KSHV is now undergoing strong selection and is in the process of adaptation.

**Supplementary Information:**

The online version contains supplementary material available at 10.1007/s00705-025-06259-9.

## Introduction

Kaposi’s sarcoma-associated herpesvirus (KSHV) (species *Rhadinovirus humangamma8*), also known as human gammaherpesvirus 8, is a member of the genus *Rhadinovirus*, subfamily *Gammaherpesvirinae*, and family *Orthoherpesviridae* that is distributed predominantly in sub-Saharan Africa and, to a lesser extent, in the Mediterranean region and is found less frequently in other parts of the world [1]. In most cases, individuals infected with KSHV are also infected with human immunodeficiency virus (HIV), and KSHV patients frequently belong to certain ethnic or behavioral groups, especially in developed countries [2]. KSHV is an opportunistic pathogen that does not usually cause symptoms in healthy humans. However, it can cause Kaposi’s sarcoma (KS) in immunodeficient people, such as the elderly or those infected with HIV, and sometimes causes primary effusion lymphoma and multicentric Castelman disease [2]. KSHV is thought to have co-evolved with humans because of its worldwide dispersal and high prevalence in certain ethnic communities of sub-Saharan Africa [3].

KSHV has a 140-kbp double-stranded DNA genome containing at least 81 open reading frames (ORFs) [4]. ORF-K1 is a transmembrane protein with a length of 289 amino acids that is encoded by the first ORF in the genome. It is unique to KSHV and has no other protein homologs [5]. The K1 gene is commonly used as a genetic marker to identify subtypes of KSHV [6, 7]. Phylogenetic analysis based on the K1 gene has shown that KSHV is divided into six main subtypes, A–F, of which A–C are currently the most prevalent worldwide [8–10].

Despite the use of the K1 gene as a genetic marker for subtyping, little is known about the rate and mode of its evolution. In a previous study, a dataset of 550 K1 gene sequences from different countries was used for phylogenetic analysis to examine the geographical distribution of KSHV [11]. In another study, the complete genomes of KSHV isolates from Cameroonian KS patients were sequenced to explore the association between different subtypes of KSHV sequences and disease occurrence [12]. Extensive investigations have been conducted into the diversity and sequence variations of the K1 gene. However, the recombination and selection history of this gene remains to be elucidated.

In this study, to investigate the evolutionary history of KSHV, we carried out an evolutionary biological analysis of the K1 gene, which revealed that recombination events, particularly between subtypes A and C, have played a significant role in the evolutionary history of KSHV.

## Materials and methods

### Data preparation, quality control, and sequence alignment

We downloaded 2157 ORF-K1 sequences from the GenBank database. In the first screening, K1 genes that were marked as “nonfunctional” or “synthetic construct clone” in the GenBank annotation were removed from the dataset. We then calculated the GC content of each sequence and removed sequences with a GC content greater than 50%. We also screened K1 sequences by sequence length. A typical K1 open reading frame is about 870 nt in length, but many sequences in our dataset were less than half of this length. Screening based on length resulted in an “all_long” dataset, containing 1304 sequences exceeding 400 nt in length, and this was used in the current study. Sequences were aligned at the codon level using MAFFT (v7.294b) software [13] and PRANK software [14].

### Phylogenetic analysis and subtyping

A maximum-likelihood tree based on nucleotide sequences was made using IQ-TREE software with 1000 bootstrap replicates (Supplementary Fig. S1). As no outgroup homologous to K1 was available in the NCBI database, no outgroup was included. SYM+R4 was selected as the best model by IQ-TREE [15, 16]. For the visualization and presentation of the phylogenetic tree, we used FigTree and the ggtree package in R. Using sequences with subtype information from previous publications as guide sequences, we assigned subtypes to all of the K1 sequences, as shown in Supplementary Figure S1. Details regarding the sampling regions, collection times, subtypes sampled, and updated subtypes are summarized in Supplementary Table S1. The subtype designation “N0” indicates that no subtype information was available. Sequences that were difficult to assign to any subtype were excluded from the subsequent analysis.

### Sampling locations and dates

The sampling location and sampling date for each sequence was obtained based on published articles and annotations in the GenBank entries. Information about the sampling location was obtained for most of the sequences, but many were lacking the sampling date. Out of 2157 sequences, only 891 provided precise sampling dates. If the sampling date was not reported in the referenced article, we used the GenBank registration date instead, and these sequences are marked with an “s” before the date label in our database. Accession numbers and information for each sequence are summarized in Supplementary Table S1.

### Median-joining network analysis

In order to construct a median-joining network based on nucleotide sequence haplotypes, 603 incomplete sequences among the 1304 sequences in the dataset were removed, leaving a total of 701 K1 sequences without any missing data, which were selected for haplotype identification. Haplotypes of K1 were identified using DnaSP software version 5.10.1 [17], and a median-joining network was constructed using Network (v5) software [18].

### Recombination of K1

The recombination rate was inferred by counting the segregating sites (N) and haplotypes (M) at the nucleotide level using a dataset of 701 sequences. If M > N, M - N is the minimum number of recombination events that can explain the data, which can be regarded as a minimum boundary for the recombination rate [19]. A further test of recombination of K1 was performed using RDP4 software, using all seven methods included in the software package: RDP [20], GENECONV [21], Bootscan [22], Maxchi [23], Chimaera [24], SiSscan [25], and 3seq [26]. If a likely recombination event was detected by three or more of the seven methods, we counted it as a positive result. Recombination breakpoints and the sequences involved in the event were also recorded. The specific sequences associated with recombination events and the corresponding validation methods are shown in Supplementary Table S2.

### Genetic drift of K1

We used TreeMix software v1.13 [27] to infer the introgression history among locations and subtypes of K1. A dataset of 1024 sequences was obtained by filtering sequences with fewer than 50 missing bases. We then transferred the K1 alignment to a multiple VCF file using SNP-sites software [28]. Populations were distinguished by considering both sampling location and subtype. For example, sequences of the A subtype in Africa were designated as the “AF_A” population. In this way, the 1024 sequences were divided into 27 populations.

We applied TreeMix to reconstruct the population mixture history of K1, testing from one to 15 possible migration events. Covariance for each migration event was used to quantify the model fit. In the recombination test, we detected 11 major recombination events, and we therefore think that the number of actual migration events should be larger than 11. Fig. 3 shows a gene flow diagram assuming fewer than 13 migration events.

### Bayesian skyline plot

Of the 2157 sequences, 891 had precise information about the sampling time, including representatives of each of the subtypes A–F. Considering the limited computational capabilities of BEAST for handling large data sets, we further refined our dataset by selecting 343 sequences (<50 missing bases) that had fewer gaps than the others. We used these 343 sequences to calculate historical population size changes using BEAST v1.10.4 software [29, 30]. A model test using MEGA software identified the JC69+G model as the best fit. A clock analysis was performed using the baseml module in PAML [31], and the results indicated that the non-clock model was significantly better than the clock model. Therefore, we selected the JC69+G model with the assumption of independent rate variation across lineages (the uncorrelated relaxed clock model) [32]. For each subtype, we used all 891 sequences in the dataset to test for the corresponding subtype.

For the A subtype, the convergence of MCMC processes was poor, irrespective of the length of time the chain was run. We found that this was due to a subpopulation division within the A subtype, namely that A0–A4 and A5 differed in their evolutionary rates. We therefore separated the A subtype into two subgroups, A0–A4 and A5, for the calculation. The effective sample size for the analysis was larger than 200.

## Results

### Global distribution of KSHV subtypes

**Fig. 1:**
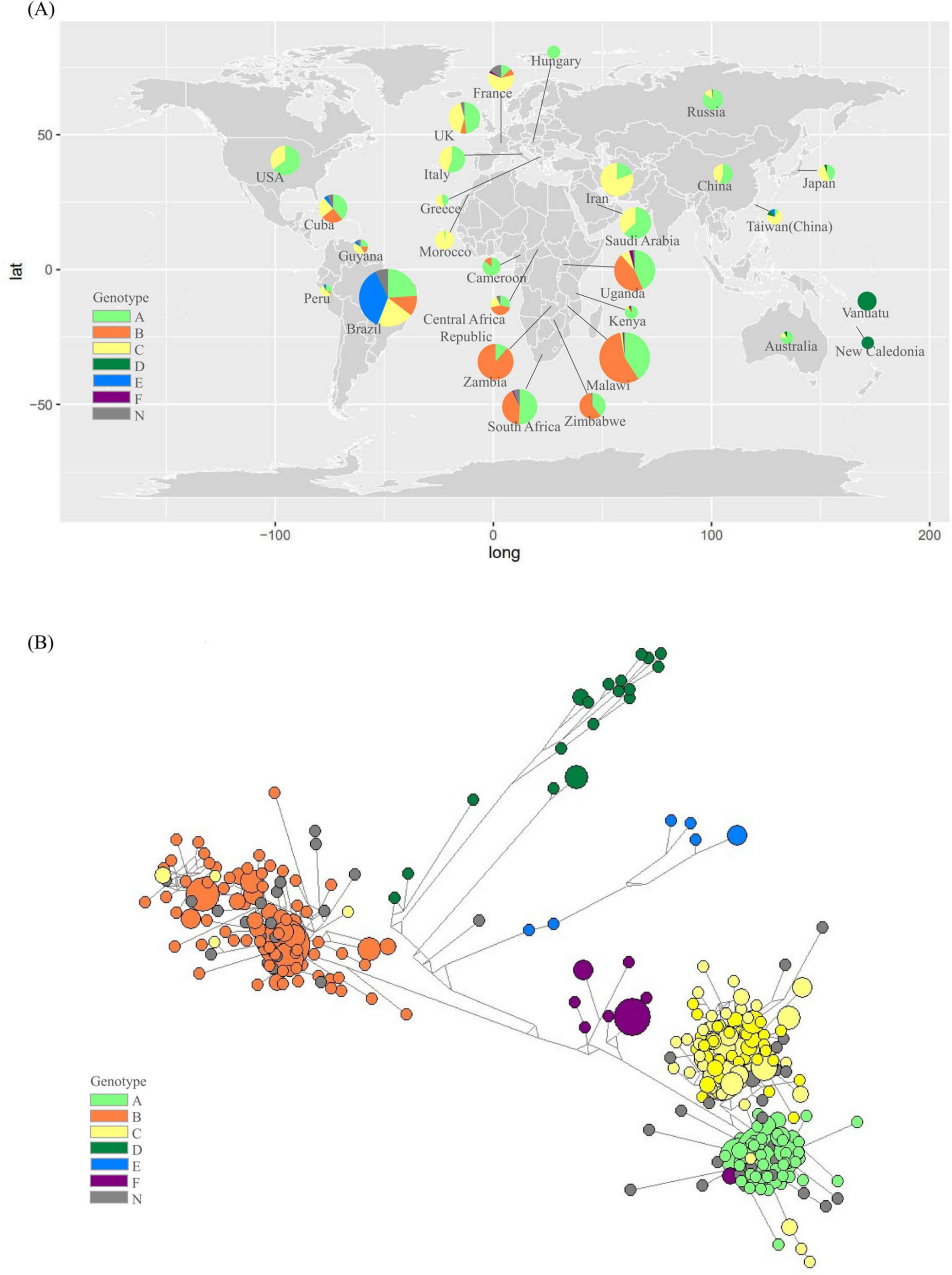
Global distribution of KSHV subtypes. (A) Summary of sampling locations and subtypes of 2157 ORF-K1 genes. The number of sequences in each region is represented by a pie chart. The subtype assignment of each sequence was confirmed by phylogenetic analysis (Supplementary Fig. S1). (B) Nucleotide median-joining network of 701 ORF-K1 genes. We screened out sequences with missing data, and used 701 ORF-K1s for the calculation. Haplotypes of ORF-K1 were identified using DNASP software [17], and a median-joining network was constructed using Network software

We collected 2157 ORF-K1 gene sequences from 47 countries or territories up to 2022, encompassing all continents except Antarctica. We initially aligned the collected sequences, and the results included the two hypervariable regions of K1. Subsequently, we identified subtypes of KSHV based on phylogenetic analysis of ORF-K1 genes. (Supplementary Fig. S1, Supplementary Table S1), and this showed that our data covered all of the major subtypes of KSHV (A to F) (Fig. 1A). The number of sequences in each region is shown by a pie chart. Sequences that could not be unambiguously classified into any of the subtypes A–F were designated as “N”. To investigate the evolutionary relationship of the KSHV isolates, we conducted a haplotype network analysis using 701 ORF-K1 gene sequences. Median-joining networks were computed at the nucleotide level (Fig. 1B) [18].

As shown in Figure 1A, subtype A was found to be the most prevalent worldwide (830, 38.4%), followed by subtype B (593, 27.4%) and subtype C (578, 26.7%). Subtypes E (74, 3.4%), D (55, 2.5%), and F (20, 0.9%) had lower prevalence rates. The largest number of sequences originated from Africa (894, 41.4%), followed by South America (359, 16.6%), Asia (348, 16.1%), Europe (329, 15.2%), North America (164, 7.6%), and Oceania (63, 2.9%). These results are consistent with the distribution of 550 ORF-K1 sequences reported by Lopes et al. [11], with slight variations, as discussed in the following sections.

### Recombination history

**Fig. 2:**
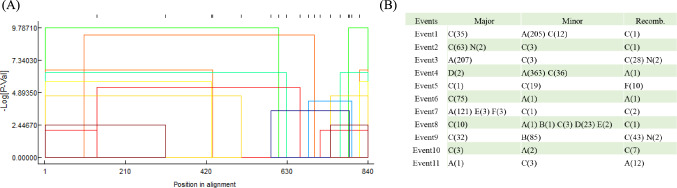
Recombination events in ORF-K1. (A) Breakpoint distribution plot for all detected recombination events. The *x*-axis represents amino acid positions in ORF-K1, and the *y*-axis is the negative log-transformed *P*-value for recombined sequences and their parent sequences, calculated using RDP4 software. (B) Summary of subtypes involved in each recombination event. Numbers in parentheses are the number of sequences involved in each event, as parents (Major or Minor) or as recombinants (Recomb.)

We estimated the frequency of recombination events occurring within K1 by computing the difference between the number of haplotypes (M) and segregating sites (N). For recent evolution (within a single species), if M > N, M - N is the minimum number of recombination events that can explain the data [19]. We identified 336 haplotypes with 239 segregating sites using DnaSP software (Fig. 1B), resulting in a minimum average recombination rate of 0.41 per site, indicating that recombination has occurred frequently in ORF-K1. We conducted a further recombination test using RDP4 software [33] (Fig. 2A and B, Supplementary Table S2). The data we used for this test comprised 1304 ORF-K1 sequences longer than 400 nt. Eleven major recombination events were identified by three or more of the seven recombination detection methods. Figure 2A shows the distribution of the breakpoints of all of the detected recombination events. Notably, the breakpoints were almost evenly distributed across all alignments, and no specific recombination hotspots were detected. RDP4 identified 901 recombination events among the 1304 ORF-K1 sequences, either as donors (the source of recombinant sequences) or recombinants. All major lineages of ORF-K1, including subtypes A–F, were found to have undergone one or more recombination events. Notably, subtype A included 563 sequences (43.2%), and subtype C included 295 sequences (22.6%), both of which were involved in nearly all of the reported recombination events. Conversely, subtype B, with a total of 344 sequences (26.4%), participated solely as a minor parental sequence in events 8 and 9. This observation underscores the high homogeneity between subtypes A and C, while subtype B appears relatively independent in its evolutionary history, consistent with the nucleotide haplotype results shown in Figure 1B.

### Direction of gene flow

**Fig. 3:**
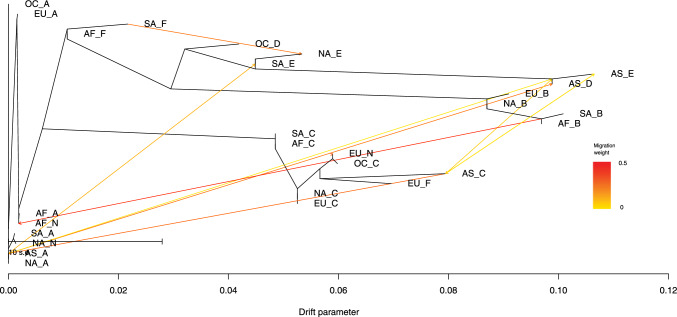
Gene flow of ORF-K1. Population mixture history of ORF-K1, determined using TreeMix Software. Continents are represented by abbreviations: AF, Africa; AS, Asia; EU, Europe; NA, North America; OC, Oceania; SA, South America. The *x*-axis represents the drift parameter. The arrows connecting populations illustrate migration weights, with a closer approach to the red color indicating a greater migration weight, which refers to the relative contribution of genetic material from one subtype to another

We then examined the direction of flow of the ORF-K1 gene using TreeMix software [27] (Fig. 3). Populations of ORF-K1 gene are identified by both sampling location and subtype/clade. For example, AF_A means subtype A sampled from Africa.

Our analysis revealed that most of the gene flow events occurred within continents. The A-F subtypes formed distinct monophyletic groups. Notably, subtypes A and C exhibited smaller drift parameters, implying a higher level of gene flow and admixture. In contrast, subtype B displayed a larger drift parameter, indicative of a greater degree of genetic differentiation. The relationships are also consistent with those shown in Figure 1B.

### Coalescence times and ancestral demography

We estimated the time to the most recent common ancestor (tMRCA) of the ORF-K1 genes of subtypes A–F. In order to use the sampling times of the sequences as calibration points, only the 891 sequences with a precise sampling time were included in the analysis. To decrease the amount of computing capacity required, the dataset was reduced to 343 sequences for the calculation.

**Fig. 4:**
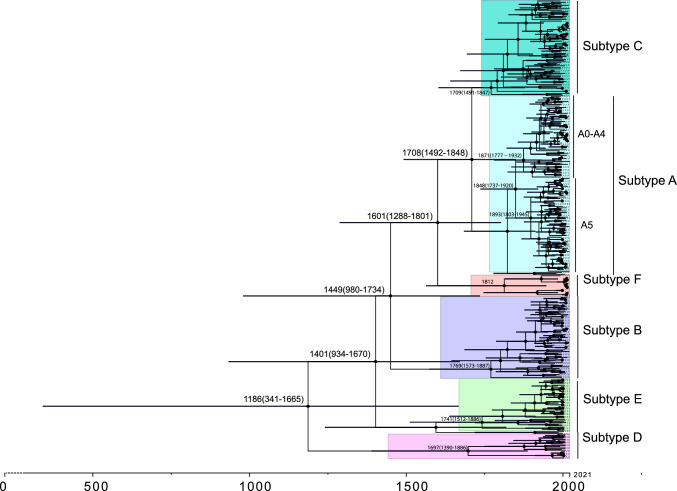
Time tree of KSHV subtypes. Horizontal bars spanning nodes represent the 95% confidence interval of the estimated time of root node. Unlike the tree shown in Supplementary Figure S1, the MCC tree shows the time of each root node along with its 95% confidence interval. Distinction of different subtypes is based on the common ancestral branches in the phylogenetic tree

**Fig. 5:**
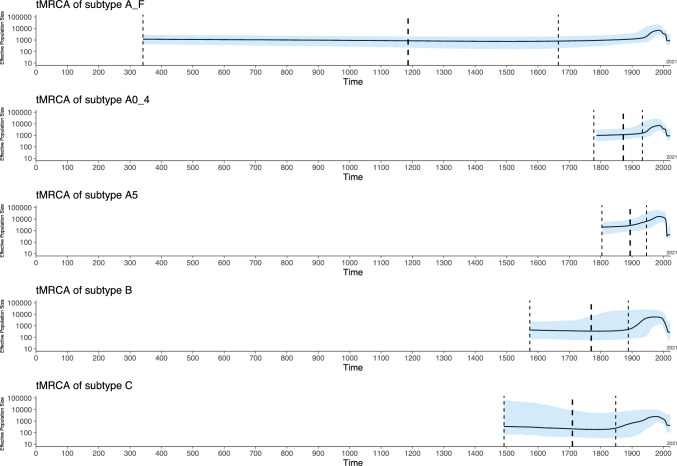
Historical population size changes of KSHV subtypes. Historical effective population size changes though time are shown for subtypes A–F or just the three major ORF-K1 subtypes. We separated the A subtype into A0–A4 and A5 based on differences in their evolutionary positions in the phylogenetic tree (see Supplementary Figure S1). The blue lines indicate the 95% confidence interval for the determination of the tMRCA

The most recent common ancestor of subtypes A–F was estimated to date to the year 1186, with the 95% confidence interval (CI) ranging from 341 AD to 1665 AD (Fig. 4). For the three globally prevalent KSHV subtypes, A0-A4, it was 1871 AD (95%CI: 1777–1932 AD), for A5 it was 1893 AD (95%CI: 1803–1945 AD), for B it was 1769 AD (95%CI: 1573–1887 AD), and for C it was 1709 AD (95%CI: 1491–1847 AD) (Fig. 5). These results suggest that the emergence and spread of the major KSHV subtypes occurred within the past 300 years. The evolutionary rate of ORF-K1 is 1.55×${10}^{-4}$ (95%CI: 8.44×${10}^{-5}$ - 2.33×${10}^{-4}$) per site per year. In comparison, the complete genome sequences of other dsDNA viruses, such as variola virus (VARV) with an evolutionary rate on the order of 10^−6^/site·year, and herpes simplex virus 1 (on the order of 10^−7^/site·year), have much slower mutation rates [34], suggesting that KSHV subtypes are undergoing intense selective pressure. The effective population size of ORF-K1 increased during the 1960s, possibly due to the increase in the global human population size that occurred after World War II. In 1996, potent HIV protease inhibitor drugs became widely available, and as a result, the occurrence of AIDS-related opportunistic infections decreased markedly [35].

## Discussion

In this study, we constructed a phylogenetic tree of the KSHV K1 gene by collecting a total of 2157 ORF-K1 sequences. For K1 sequences lacking subtype information, we assigned new subtypes based on their positions in the phylogenetic tree (Supplementary Table S1). In addition, we compiled information about the global epidemiological status of KSHV. According to the World Cancer Report published by the World Health Organization (WHO) in 2020, the prevalence of KSHV infection is high in sub-Saharan Africa (50–95%), intermediate in Mediterranean countries (10%), and generally lower in other parts of the world. Age-standardized incidence rates indicate that KS has a remarkably high incidence in southern Africa, with a rate of up to 8.5 per 100,000 individuals, while southern Europe and South America have rates of 0.8 per 100,000 and 0.7 per 100,000 individuals [36].

We observed that a significant proportion of K1 gene sequencing studies have been conducted in Africa, accounting for nearly half of the studies, followed by South America (17%), Asia (16%), and Europe (15%). This distribution aligns with the current epidemiological status of KSHV. It is worth noting that African populations exhibit a high seroprevalence of KSHV and possess substantial genetic diversity. Existing research has predominantly focused on the evolutionary characteristics of KSHV and its association with KS incidence in specific African countries. However, there has been a scarcity of large-scale studies at the gene level for elucidating the variation and recombination patterns of KSHV during the evolutionary process.

In the phylogenetic tree depicted in Supplementary Figure S1, we have noted several instances of considerable temporal gaps between sequences that belong to the same monophyletic cluster. For instance, sequences MW892534.1 and AY0042954.1 were sampled in 1999 and 2019, respectively. This contrasts with the behavior of ancient viruses such as SARS-CoV-2 or influenza virus [37, 38]. Unlike those viruses, KSHV sequences do not form clusters near the root node and do not exhibit a stepping-stone evolutionary pattern. This observation suggests, to some extent, that KSHV is a more recently emerged virus. This phenomenon could be attributed to the persistence of KSHV within small populations, which aligns with the phylogenetic patterns observed in endemic infectious diseases [39]. In the median-joining networks we constructed, certain nucleotide haplotypes were found to have been misclassified. For instance, MZ923819.1 (Hap_299) and MZ923820.1 (Hap_300) were annotated as subtype C1 in the NCBI database, while phylogenetic analysis correctly categorizes them as subtype A and B, respectively. This pattern is consistent with several other misclassified subtypes we have identified.

In our analysis of recombination and gene flow in the K1 gene, we observed that recombination events between subtypes A and C are frequent, accompanied by a higher frequency of gene flow and admixture. In contrast, subtype B exhibits a much lower rate of involvement in recombination and has a higher drift parameter. We also observed only minimal gene flow between continents, with a rare instance of transfer from the South American F subtype (SA_F) to the North American E subtype (NA_E). This phenomenon may be attributed to the K1 sequences sampled, which have undergone recombination and evolution over a considerable period, rather than representing recently introduced K1 genes on a specific continent. Further investigation with a larger and more diverse set of samples, possibly utilizing novel methodologies, is needed to assess the gene flow of K1 more accurately.

Another intriguing finding in this study is the relatively recent common ancestor of the ORF-K1 gene (from around 1186 AD), which contrasts with the general idea that KSHV is an ancient virus that has co-evolved with humans. In the 12th and 13th centuries, the largest-scale historical events of the Crusades occurred, resulting in population movement and trade between Europe, Asia, and Africa [40]. At that time, the Solomon dynasty in East Africa began exporting ivory, rhino horn, turtle shells, spices, gold, and slaves to neighboring countries through the Red Sea route, while the Mali Empire in West Africa vigorously developed commerce through the Mediterranean. Furthermore, during the 12th century, the flourishing Silk Road of the Tang Dynasty in China and subsequently the Maritime Silk Road of the Song Dynasty facilitated extensive cultural and population migration [41]. This coincidence between the tMRCA of KSHV and modern historical events suggests that KSHV spread worldwide during the 12th century. Compared to leprosy, which was prevalent at that time, KSHV is a more temperate pathogen, causing Kaposi’s sarcoma only when the host’s immune system is compromised. Although leprosy was once incurable in the era of limited medical technology, with the advent of leprosy vaccines, the disease is now only found in a few remote regions with inadequate health and medical care [42]. This may explain the coexistence of KSHV with modern humans for several centuries.

Our study has several limitations, with the most significant being that it is not known whether the K1 sequence results can be extrapolated to the full KSHV genome. Currently, there is limited research on the full genome of KSHV, and it is challenging to obtain enough sequences for evolutionary analysis. Here, we used K1 gene sequences to investigate the evolution of different KSHV subtypes, but we intend to use full-genome sequences for evolutionary analysis of KSHV in future studies.

In conclusion, we compared a large number of KSHV K1 gene sequences from diverse geographic origins, providing insights into the global distribution of KSHV subtypes and shedding light on the intricate recombination and selection dynamics of the ORF-K1 gene. Our findings enhance our understanding of the adaptation and evolution mechanisms of KSHV, offering valuable references for epidemiological and clinical research and paving the way for targeted therapeutic strategies and vaccine development.

## Supplementary Information

Below is the link to the electronic supplementary material.

Supplementary file1 (PDF 207 KB)

Supplementary file2 (CSV 131 KB)

Supplementary file3 (CSV 46 KB)

## Data Availability

The data that support the findings of this study are available in the supplementary files of this article.
