# Supplementary figures and images for: Evolutionary analysis and population dynamics in the global transmission of Kaposi’s sarcoma-associated herpesvirus
Source: Arch Virol. 2025 Mar 27;170(5):92. doi: 10.1007/s00705-025-06259-9 (PMC11950004; doi:10.1007/s00705-025-06259-9)

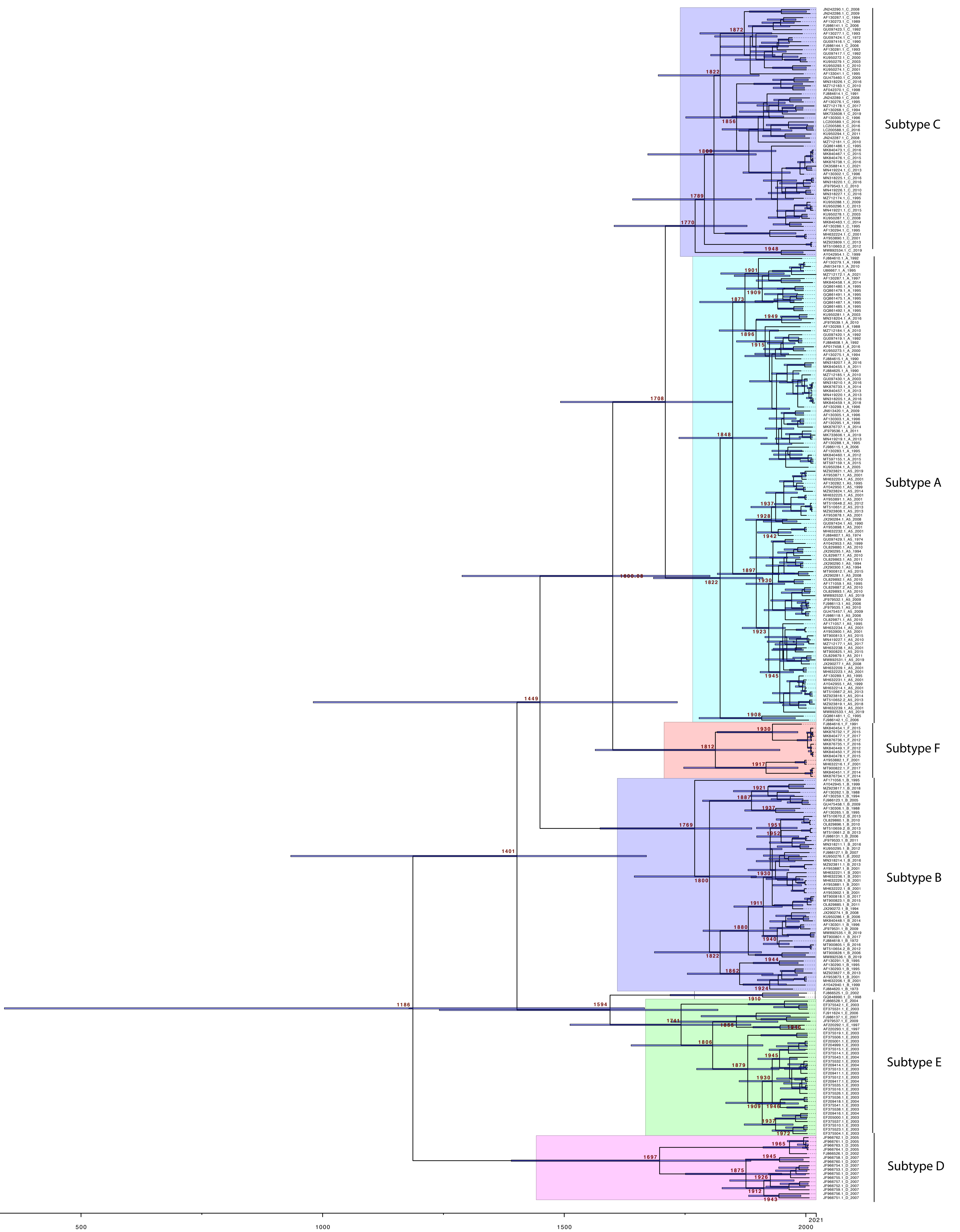

Subtype C

Subtype A

Subtype F

Subtype B

Subtype E

Subtype D

Supplement: Supplementary file 1 — Supplementary file1 (PDF 207 KB) [file 705_2025_6259_MOESM1_ESM.pdf]
